# Neonatal respiratory distress beyond common causes: surfactant dysfunction disorders and other etiologies of childhood interstitial lung disease

**DOI:** 10.3389/fped.2026.1940912

**Published:** 2026-10-09

**Authors:** Ayyüce Ünlü, Işıl Bilgiç, Meltem Kürtül Çakar, Gamze Akca Dinç, Hande Yetişgin, Çelebi Yıldırım, Sanem Eryılmaz Polat, Dilber Ademhan Tural, Gökçen Dilşa Tuğcu, Esra Karakuş, Güzin Cinel

**Affiliations:** 1 Department of Pediatric Pulmonology, Ankara Bilkent City Hospital, Ankara, Türkiye; 2 Department of Pediatric Pulmonology, Ankara Yıldırım Beyazıt University Faculty of Medicine, Ankara, Türkiye; 3 Department of Pathology, Ankara Bilkent City Hospital, Ankara, Türkiye

**Keywords:** ABCA3 gene mutation, childhood interstitial lung disease (chILD), neonatal respiratory distress, NKX2-1, surphactant protein dysfunction

## Abstract

**Background:**

Neonatal interstitial lung disease (ILD) encompasses a heterogeneous group of rare disorders, among which surfactant dysfunction disorders represent a clinically distinct and severe subgroup. Diagnosis is challenging because presentation frequently overlaps with common neonatal respiratory conditions, and classification in neonates remains particularly difficult.

**Objectives:**

To describe the clinical characteristics, genetic spectrum, and outcomes of neonatal-onset childhood interstitial lung disease (chILD), with a focus on etiology-related differences in clinical presentation between surfactant dysfunction disorders and other chILD subtypes.

**Methods:**

This retrospective study included 14 patients with neonatal-onset chILD diagnosed between 2019 and 2025. Patients underwent a standardized evaluation protocol and were stratified by surfactant dysfunction mutation status to explore etiology-related differences in clinical presentation and outcome.

**Results:**

Fourteen patients were included (50% male; 42.9% consanguineous; 50% premature). Pathogenic or likely pathogenic variants were identified in 7/14 patients (50%), most commonly in *ABCA3* (21.4%) and *NKX2-1* (14.3%). Patients with surfactant dysfunction mutations presented earlier (symptom onset <24 h in 100% vs. 77.8%) and required higher peak oxygen support (median FiO₂ 100% vs. 55%), whereas those with other etiologies had a longer but less fatal course. Pulmonary hypertension was present in 42.9%. Mortality was 21.4% after a median follow-up of 36 months.

**Conclusions:**

In this small cohort, surfactant dysfunction disorders showed numerically earlier symptom onset, higher oxygen requirements, and higher mortality compared with other etiologies, although between-group differences did not reach statistical significance. Repeated surfactant requirements or failure to wean from respiratory support should prompt early systematic clinical, radiological, and genetic evaluation, particularly in preterm infants, in whom diagnostic delay may occur due to clinical overlap with respiratory distress syndrome.

## 1 Introduction

Childhood interstitial lung disease (chILD) comprises a heterogeneous group of disorders affecting the alveolar epithelium, pulmonary interstitium, and capillary structures, resulting in impaired gas exchange and progressive respiratory insufficiency. Diagnosis is challenging because it often requires invasive or inaccessible tests, and classification in neonates remains particularly difficult (1). Diagnosis of chILD in neonates and infants remains challenging because clinical presentation is often nonspecific and diagnostic evaluation may require advanced imaging, genetic testing, bronchoalveolar lavage (BAL), or lung biopsy (2). Current classification systems for chILD in infancy include diffuse developmental disorders, alveolar growth abnormalities, surfactant dysfunction disorders, and specific disorders of infancy such as neuroendocrine cell hyperplasia of infancy (NEHI) and pulmonary interstitial glycogenosis (PIG) (3–5). Neonates with chILD commonly present with tachypnea, hypoxemia, diffuse pulmonary infiltrates, and respiratory failure, findings that may overlap with more common neonatal disorders including respiratory distress syndrome, transient tachypnea of the newborn, pulmonary infection, and bronchopulmonary dysplasia (2).

In recent years, next-generation sequencing (NGS) has become increasingly important in the evaluation of neonatal diffuse lung disease, particularly for surfactant protein dysfunction disorders associated with pathogenic variants in *SFTPB, SFTPC, ABCA3,* and *NKX2-1* (2, 6). Clinical presentation in neonatal chILD varies according to the underlying etiology. Surfactant dysfunction disorders, in particular those associated with *ABCA3* mutations, have been reported to present with early-onset severe respiratory failure and high oxygen requirements, whereas other chILD subtypes may follow a more variable course (7, 8). Despite advances in molecular diagnostics, data regarding the clinical presentation, radiological findings, molecular spectrum, and outcomes of neonatal chILD remain limited. This study aimed to evaluate the diagnostic approach, molecular findings, and clinical outcomes of neonates diagnosed with chILD at a tertiary pediatric center.

## 2 Materials and methods

### 2.1 Study design and participants

This single-center retrospective cohort study included patients with neonatal-onset chILD who were diagnosed and followed at our tertiary referral center between January 2019 and December 2025. Patients were identified from two sources: neonatal intensive care unit referrals for persistent respiratory failure and outpatient pediatric pulmonology clinic follow-ups in which respiratory symptoms had been documented during the neonatal period.

Electronic medical records were reviewed, and demographic, clinical, and radiological characteristics; personal and family history; gestational age, birth weight, 1–5. minutes APGAR (Appearance, Pulse, Grimace, Activity, Respiration) score. Parental consanguinity; treatment data; bronchoscopy findings; genetic test results; and lung biopsy findings were retrospectively analyzed.

### 2.2 Inclusion and exclusion criteria

The inclusion and exclusion criteria applied in this study have been described in detail previously (9). Briefly, cases were defined according to chILD syndrome criteria requiring fulfillment of at least three of the following four features: (1) respiratory symptoms; (2) respiratory signs (e.g., tachypnea, retractions, failure to thrive); (3) hypoxemia; and (4) diffuse abnormalities on chest radiography or computed tomography (4, 10). All patients must have had symptom onset within the first 28 days of life. Patients were excluded if they had: respiratory distress syndrome (RDS) with full and sustained clinical response to surfactant replacement by day 7 of life without evidence of residual diffuse lung disease; confirmed congenital heart disease, cystic fibrosis, primary ciliary dyskinesia, or perinatal infection as the primary etiology; or insufficient clinical data for classification. The standardized diagnostic evaluation protocol applied in this study follows our previously reported approach (9), comprising echocardiography, immunological and metabolic screening, and neurological evaluation when clinically indicated, followed by a stepwise diagnostic pathway incorporating chest imaging, genetic testing, and — when non-invasive evaluation remained inconclusive — bronchoscopy or lung biopsy (Figure 1).

**Figure 1:**
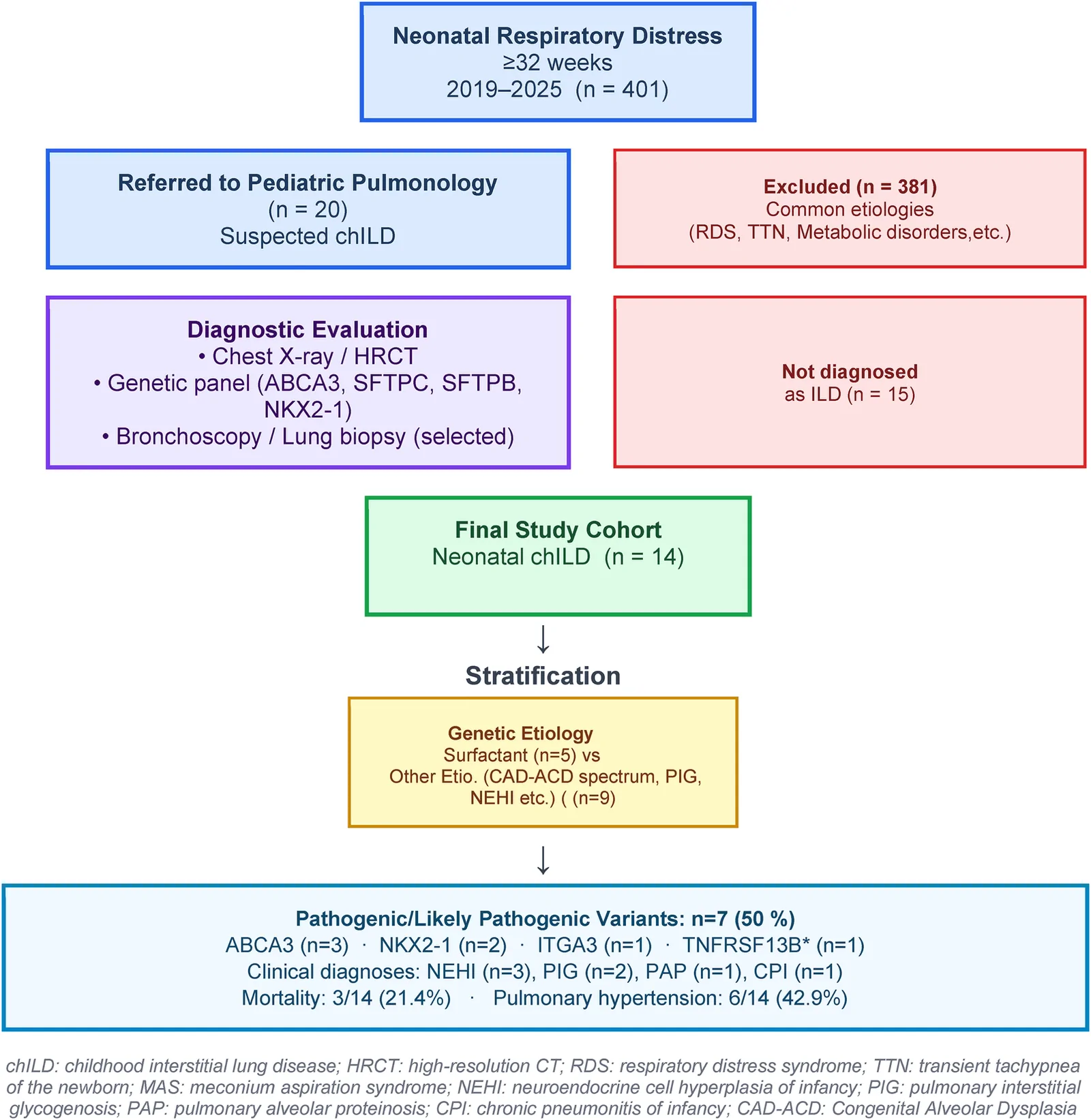
Flowchart illustrating the selection and diagnostic evaluation of 14 patients with neonatal-onset childhood interstitial lung disease. Following assessment for common neonatal respiratory disorders, patients underwent a standardized diagnostic evaluation including echocardiography, immunological and metabolic screening, chest imaging, and genetic testing.

### 2.3 Distinction from common neonatal respiratory disorders

A structured clinical approach was used to distinguish neonatal chILD from more common neonatal respiratory disorders, including respiratory distress syndrome (RDS), transient tachypnea of the newborn (TTN), and meconium aspiration syndrome (MAS). Patients were referred to the pediatric pulmonology department because of persistent respiratory failure, inability to wean from respiratory support, or repeated surfactant administration without sustained clinical improvement.

Further diagnostic evaluation was pursued in patients with one or more of the following features: (1) term or late-preterm gestation without an alternative explanation for severe respiratory disease; (2) limited or transient response to surfactant therapy; (3) prolonged requirement for invasive or non-invasive respiratory support; (4) persistent diffuse bilateral pulmonary infiltrates; or (5) family history of ILD, unexplained neonatal respiratory death, or parental consanguinity.

In addition, the absence of radiologic findings typically associated with bronchopulmonary dysplasia (BPD)—including hyperinflation, coarse interstitial/fibrotic opacities, and cystic or bubbly parenchymal changes—supported further evaluation for alternative diffuse lung disorders (11).

### 2.4 Radiological assessment

Chest CT acquisition parameters and image evaluation methods were consistent with our earlier report (9). Briefly, CT was available in 12 of 14 patients. The remaining 2 patients were too clinically unstable to undergo chest CT during the neonatal period; in both cases, the diagnosis (biallelic *ABCA3* mutations) was established on the basis of genetic testing alone. CT features assessed included ground-glass opacities, interlobular septal thickening, cystic changes, alveolar simplification, emphysematous changes, and a NEHI-compatible pattern (Figure 2) (12, 13).

**Figure 2:**
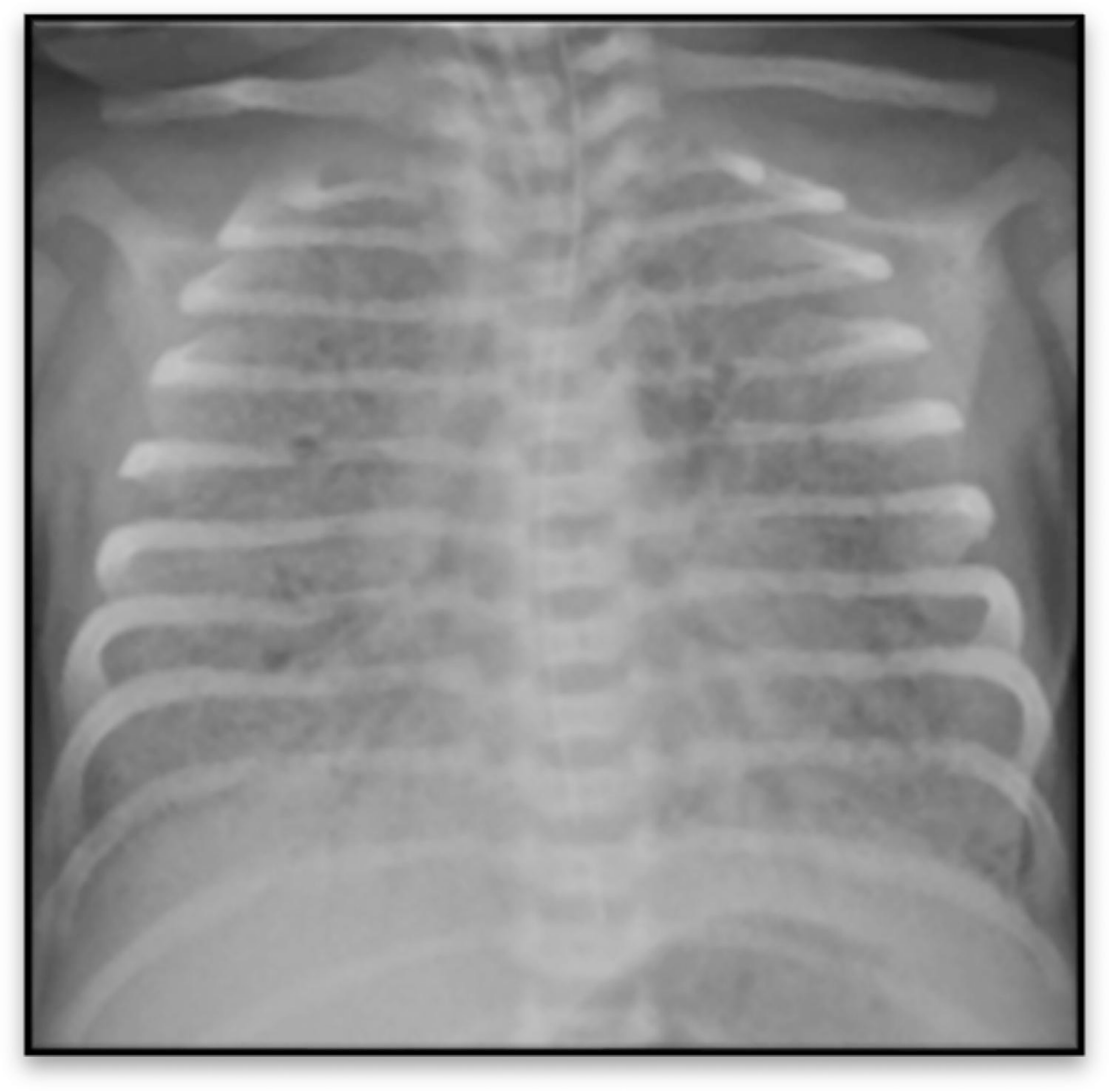
Chest radiograph of a neonate with biallelic *ABCA3* mutations demonstrating diffuse bilateral ground-glass opacities with interlobular septal thickening, mimicking the appearance of respiratory distress syndrome of prematurity. Despite repeated exogenous surfactant administration, the patient failed to achieve sustained clinical improvement, prompting escalation of diagnostic evaluation and subsequent whole-exome sequencing. ABCA3, adenosine triphosphate-binding cassette transporter A3.

### 2.5 Bronchoscopic evaluation

Flexible bronchoscopy was performed in 3 of 14 patients when clinical or radiological findings suggested a diagnosis requiring cytological confirmation or when the non-invasive workup remained inconclusive. BAL fluid was assessed macroscopically and analyzed for cell differential count, lipid-laden macrophage index, microbiological cultures, and periodic acid–Schiff (PAS) staining when clinically indicated.

### 2.6 Genetic analyses

Molecular evaluation followed a stepwise approach. A targeted gene panel encompassing surfactant protein metabolism genes (*SFTPB, SFTPC, ABCA3,* and *NKX2-1*) and genes associated with pulmonary alveolar proteinosis (*CSF2RA* and *CSF2RB*) was applied as initial testing. In patients with non-diagnostic panel results, next-generation sequencing or whole-exome sequencing was performed as indicated. All analyses were carried out at the institutional genetics laboratory. Identified variants were classified by a certified genetic counselor using the five-tier ACMG/AMP framework: pathogenic, likely pathogenic, variant of uncertain significance (VUS), likely benign, or benign (14). Variants for which available evidence was insufficient to support either pathogenicity or benignity were designated as VUS. In patients harboring such variants, the clinical and radiological phenotype was reviewed for consistency with the gene-associated disease spectrum; where concordance was observed, a possible pathogenic contribution was considered, without implying definitive causality.

### 2.7 Lung biopsy and treatment management

Surgical lung biopsy was performed in 2 of 14 patients in whom non-invasive evaluation — including clinical assessment, chest CT, and genetic testing — remained inconclusive. Biopsy specimens were processed with standard hematoxylin and eosin staining and PAS staining; the latter was used to identify glycogen-laden mesenchymal cells when pulmonary interstitial glycogenosis (PIG) was suspected (Figure 3). Histopathological interpretation was performed by an experienced pediatric pathologist.

**Figure 3:**
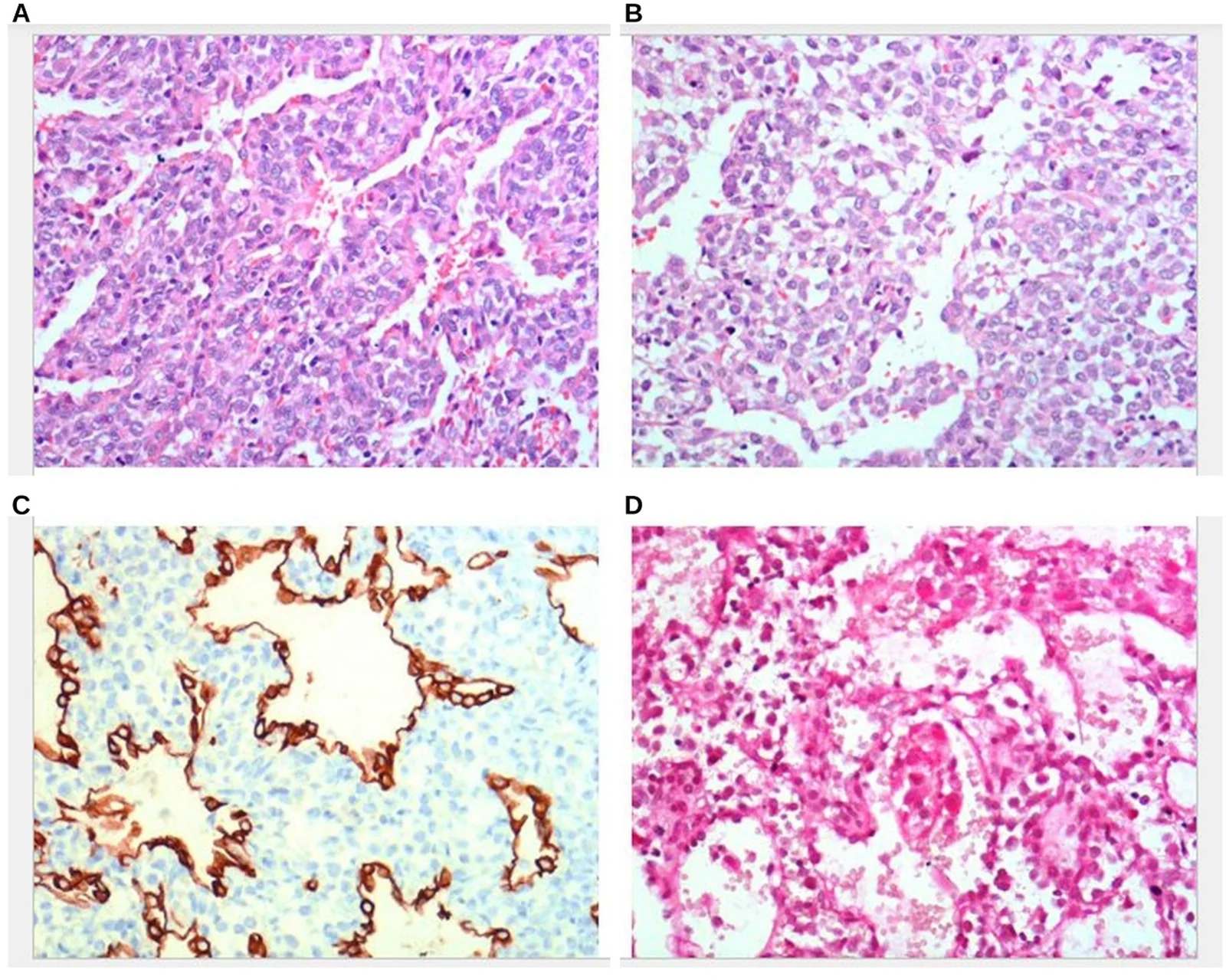
Histopathological findings of a patient diagnosed with pulmonary interstitial glycogenosis (PIG). **(A,B)** Hematoxylin and eosin staining demonstrating interstitial widening by immature-appearing mesenchymal cells with pale cytoplasm and oval nuclei, and preserved alveolar architecture without significant fibrosis or inflammation (HE, X40). **(C)** Immunohistochemical staining highlighting the distribution of interstitial cells along alveolar septa (PanCK, X400). **(D)** Periodic acid–Schiff (PAS) staining demonstrating magenta-positive cytoplasmic glycogen accumulation within interstitial mesenchymal cells, confirming the diagnosis of pulmonary interstitial glycogenosis (PAS, X400).

Pharmacological management followed published guidelines (4, 10, 15). Systemic corticosteroids (oral prednisolone 1–2 mg/kg/day or pulsed methylprednisolone 30 mg/kg/day for 3 consecutive days per month) were used as first-line therapy, with hydroxychloroquine (6–10 mg/kg/day) added in selected cases. All treatments required routine safety monitoring. Treatment response was defined as clinical improvement (weaning from supplemental oxygen or a sustained reduction in FiO_2_ requirement) together with radiological improvement on follow-up HRCT; no standardized composite response criteria currently exist for this patient population, and general management principles were adapted from existing chILD guidelines (4).

### 2.8 Patient stratification

Patients were stratified into two groups according to the presence or absence of a confirmed surfactant protein dysfunction mutation. Group 1 comprised patients with surfactant protein dysfunction mutations (*SFTPB, SFTPC, ABCA3,* or *NKX2-1*). Group 2 comprised patients with other etiologies, including NEHI, PIG, CPI, PAP, and other non-surfactant-related genetic diagnoses (*ITGA3, TNFRSF13B*). Because of the limited sample size, Group 1 was evaluated as a single group regardless of the specific surfactant gene involved. Group 2 was likewise analyzed as a single heterogeneous category (NEHI, PIG, CPI, PAP, and non-surfactant genetic variants) rather than being subdivided by specific diagnosis, to preserve statistical power for the primary comparison between surfactant protein dysfunction disorders and other etiologies.

Clinical presentation characteristics, including time of symptom onset, peak fractional inspired oxygen requirement, and duration of mechanical ventilation, were compared between Group 1 and Group 2.

### 2.9 Statistical analysis and ethics

Statistical analyses were performed using IBM SPSS Statistics version 26 (IBM Corp., Armonk, NY, USA). The normality of continuous variables was assessed using the Shapiro–Wilk test. Non-normally distributed continuous variables were analyzed using the Mann–Whitney U test and are presented as median (Q1–Q3). Categorical variables are presented as frequencies and percentages. Fisher's exact test or the chi-square test was applied as appropriate. A *p*-value of <0.05 was considered statistically significant.

The study was approved by the institutional ethics committee (Approval number: [1-25-1999]), and the requirement for informed consent was waived owing to the retrospective study design. The study was conducted in accordance with the principles of the Declaration of Helsinki.

## 3 Results

The cohort included 14 patients (50% male, 42.9% consanguineous). Prematurity was present in 7/14 (50%) cases. The median birth weight was 2922.5 g (Q1–Q3: 2285–3232.5). The median APGAR scores were 7 (Q1–Q3: 6–8) at 1 min and 9 (Q1–Q3: 7.5–9) at 5 min, indicating generally preserved early neonatal adaptation. All 14 patients required admission to the neonatal intensive care unit (NICU) during the neonatal period. 12/14 (85.7%) patients presented immediately after birth with respiratory distress characterized by tachypnea and cyanosis. The remaining 2/14 (14.3%) patients developed rapid breathing and cyanosis within the first 20 days of life and were subsequently admitted to the NICU (Table 1).

**Table 1:** Demographic, clinical, radiological, and outcome characteristics of the overall cohort and by surfactant dysfunction mutation status.

Variable	All (*n* = 14)	Group 1 (*n* = 5)	Group 2 (*n* = 9)	*p*
Demographic and perinatal characteristics				
Age, months	48 (9; 72)	60 (1.75; 72)	42 (11.5; 75)	1.00
Follow-up duration, months	36 (3.5; 69)	60 (1; 72)	24 (5; 75)	1.00
Birth weight, kg	2.9 (2.3–3.2)	2.9 (2.9–3.3)	3.2 (2.7–3.2)	0.432
Male sex, *n* (%)	7 (50%)	1 (20.0%)	6 (66.7%)	0.091
Prematurity, *n* (%)	7 (50%)	2 (40.0%)	5 (55.6%)	1.00
Antenatal steroid exposure, *n* (%)	4 (28.6%)	1 (20.0%)	3 (33.3%)	1.00
Consanguinity, *n* (%)	6 (42.9%)	4 (80.0%)	2 (22.2%)	0.091
APGAR score at 1 min	7 (6; 8)	8 (7; 8)	7 (5.5; 7.5)	0.905
APGAR score at 5 min	9 (7.5; 9)	9 (8.5; 9)	9 (7.5; 9)	1.00
Respiratory distress <24 h, *n* (%)	12/14 (85.7%)	5/5 (100%)	7/9 (77.8%)	1.00
Respiratory support				
Oxygen therapy duration, days	95 (21.5; 341.8)	55 (30; 119)	180 (120; 630)	0.438
Peak FiO₂, %	55 (30; 100)	100 (65; 100)	55 (42.5; 57.5)	0.400
Mechanical ventilation duration, days	20 (11; 62)	20 (12.5; 37.5)	55 (34.5; 75.5)	0.164
Home oxygen therapy, *n* (%)	7/14 (50.0%)	3/5 (60.0%)	4/9 (44.4%)	1.00
Pulmonary hypertension, *n* (%)	6 (42.9%)	2 (40.0%)	4 (44.4%)	1.00
Imaging findings, *n* (%)				
CXR:				
Diffuse infiltrates	14 (100%)	5/5 (100%)	9/9 (100%)	N/A
Hyperinflation	5 (35.7%)	1 (20.0%)	4 (44.4%)	0.580
Non-specific infiltrates	4 (28.6%)	1 (20.0%)	3 (33.3%)	1.00
HRCT				
Ground-glass opacities	5/12 (41.7%)	1/3 (33.3%)	4/9 (44.4%)	1.00
Septal thickening	10/12 (83.3%)	3/3 (100%)	7/9 (77.8%)	1.00
Cystic changes	6/12 (50.0%)	1/3 (33.3%)	5/9 (55.6%)	1.00
Alveolar simplification	1/12 (8.3%)	0/3 (0%)	1/9 (11.1%)	1.00
Clinical outcomes and Treatments, *n* (%)				
Alive at last follow-up	11 (78.6%)	3 (60.0%)	8 (88.9%)	0.505
Mortality	3 (21.4%)	2 (40.0%)	1 (11.1%)	0.505
Surfactant therapy	11/14 (78.6%)	4 (80.0%)	7 (77.8%)	1.00
Systemic steroid therapy	9/14 (64.3%)	4 (80.0%)	5 (55.6%)	0.580
Hydroxychloroquine therapy	3/14 (21.4%)	1 (20.0%)	2 (22.2%)	1.00

CXR, chest x-ray; HRCT, high-resolution computed tomography; FiO₂, fraction of inspired oxygen; APGAR, Appearance, Pulse, Grimace, Activity, Respiration. —: not compared between groups. Continuous variables are presented as median (Q1; Q3); categorical variables as *n* (%). Fisher's exact test or Mann–Whitney *U*-test applied as appropriate. Group 1: surfactant protein dysfunction disorders (ABCA3, NKX2-1, SFTPB, SFTPC). Group 2: other etiologies (NEHI, PIG, CPI, PAP, ITGA3, TNFRSF13B).

During the initial neonatal hospitalization, all patients demonstrated diffuse infiltrates on chest radiography (Figure 2), and hyperinflation was observed in 5 patients (35.7%). 11/14 patients (78.6%) received repeated endotracheal surfactant therapy due to persistent respiratory failure, inability to wean from mechanical ventilation, and sustained high fractional inspired oxygen (FiO₂) requirements, prompting further advanced diagnostic evaluation. The median duration of oxygen therapy was 95 days (Q1–Q3: 21.5–341.8), and the median duration of mechanical ventilation was 20 days (Q1–Q3: 11–62).

Referral to pediatric pulmonology department occurred later in preterm than term infants (median 90 vs. 57 days, *n* = 7 each; *p* = 0.442).

Chest CT was available in 12 of 14 patients. The most frequent radiologic finding was septal thickening (83.3%), followed by cystic changes (50.0%). Three of 14 patients demonstrated CT findings consistent with NEHI (Figure 4).

**Figure 4:**
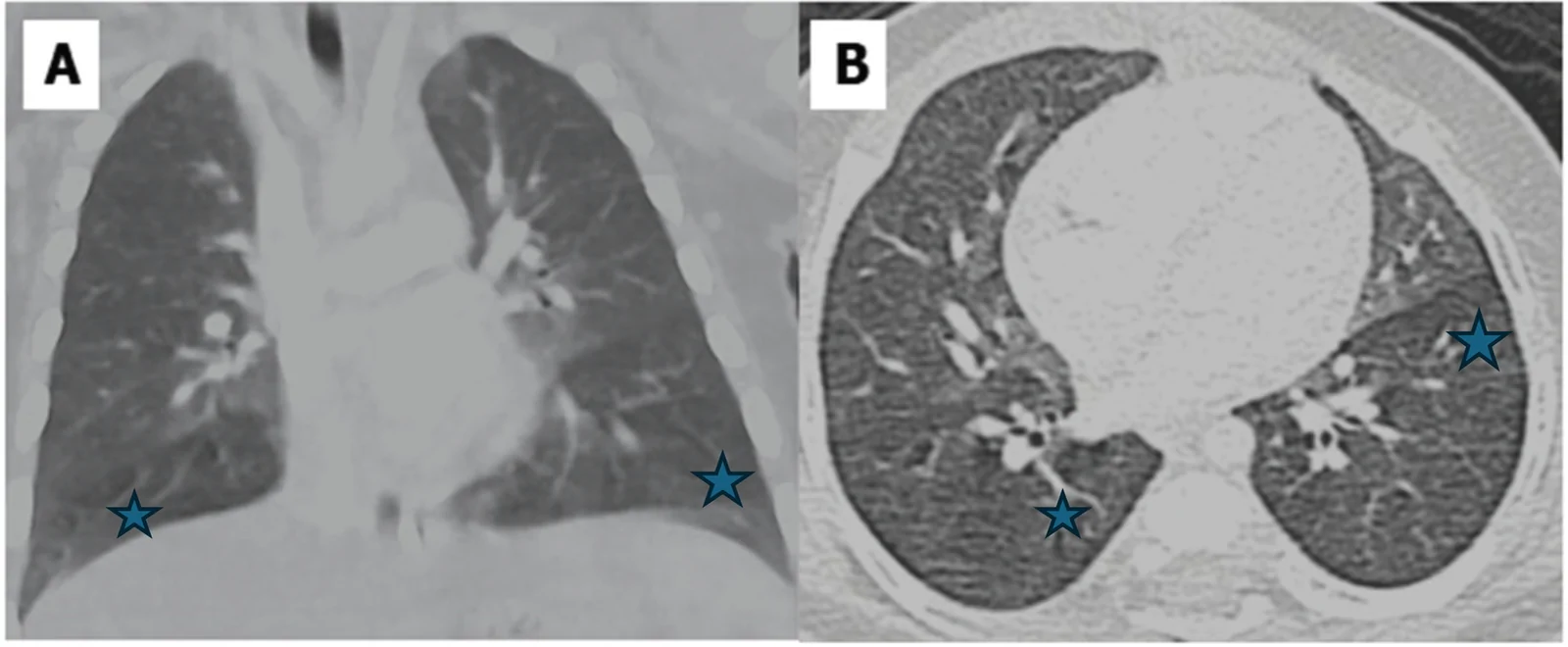
Coronal **(A)** and axial **(B)** chest CT images demonstrating bilateral ground-glass opacities with predominant involvement of the right middle lobe and lingula, compatible with neuroendocrine cell hyperplasia of infancy (NEHI).

Flexible bronchoscopy was performed in 3/14 patients. In one case, BAL revealed a characteristic milky appearance (Figure 5), leading to the diagnosis of PAP. Subsequent whole-exome sequencing identified a heterozygous variant of uncertain significance (VUS) in CSF2RA.

**Figure 5:**
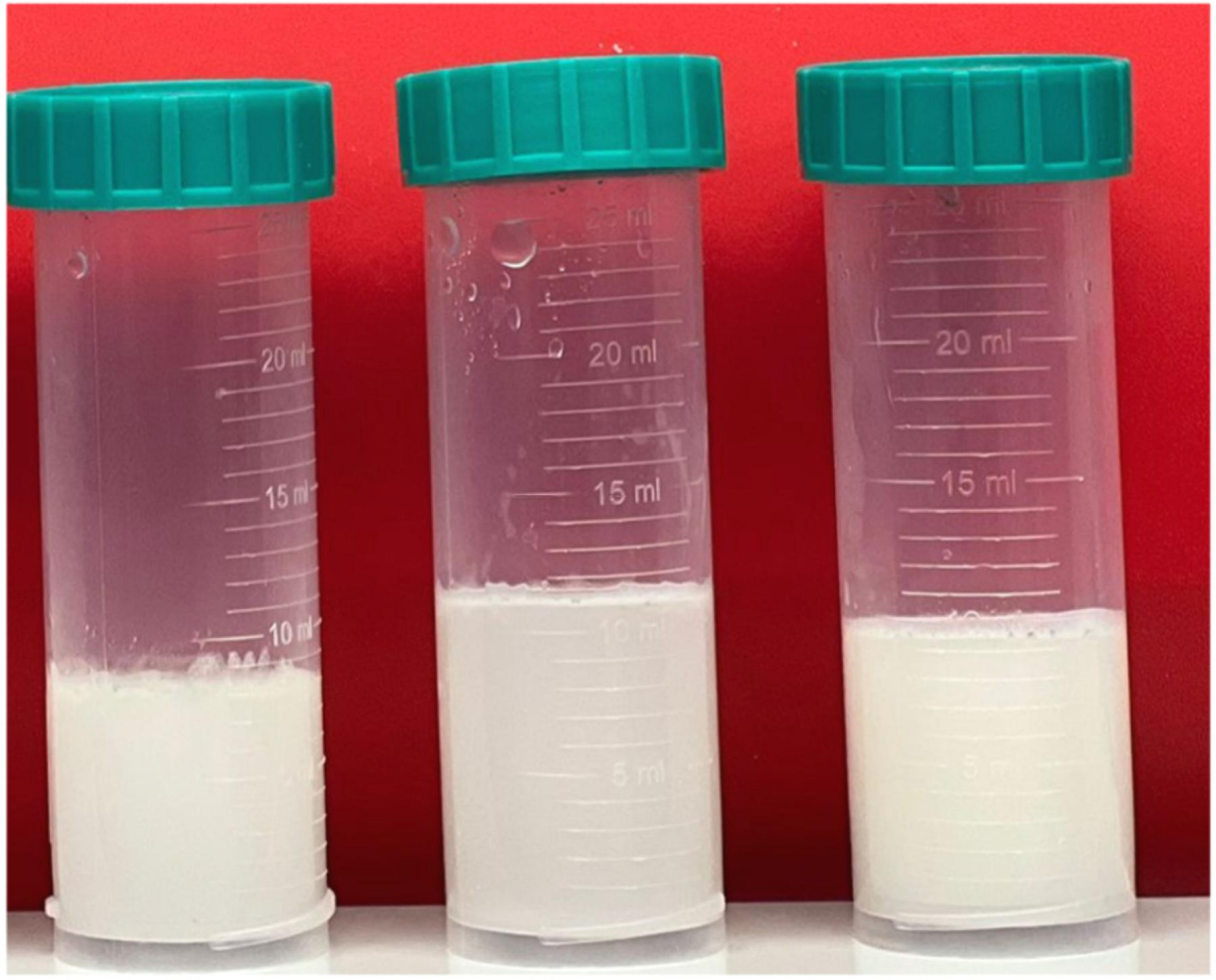
Bronchoalveolar lavage (BAL) fluid appearance in a neonate subsequently diagnosed with pulmonary alveolar proteinosis (PAP). The lavage fluid demonstrates a characteristic milky, opaque appearance due to the intra-alveolar accumulation of lipoproteinaceous surfactant material. This macroscopic finding, in conjunction with diffuse bilateral ground-glass opacities on chest imaging and the absence of an infectious etiology, prompted targeted genetic evaluation for *CSF2RA* variants. PAP, pulmonary alveolar proteinosis; CSF2RA, colony-stimulating factor 2 receptor subunit alpha.

Comprehensive genetic testing was performed in all patients. Surfactant protein gene testing was performed in all patients as first-line molecular evaluation, and whole-exome sequencing was additionally applied in five patients in whom panel testing was non-diagnostic or inconclusive. Pathogenic or likely pathogenic variants were identified in 7/14 patients (50%), including *ABCA3* mutations in 3/14 (21.4%), NKX2-1 mutations in 2/14 (14.3%), ITGA3 mutations in 1/14 (7.1%), and TNFRSF13B mutations in 1/14 (7.1%). In 2/14 patients with inconclusive noninvasive evaluation, surgical lung biopsy was diagnostic, revealing chronic pneumonitis of infancy (CPI) in one and pulmonary interstitial glycogenosis (PIG) in the other (Figure 3). Final diagnoses included NEHI in 3 patients (21.4%), PIG in 2 (14.3%), PAP in 1 (7.1%), and CPI in 1 (7.1%).

When patients were stratified into Group 1 (surfactant protein dysfunction disorders) and Group 2 (other etiologies) (Table 1), demographic and perinatal characteristics, treatments, and clinical outcomes did not differ significantly between groups (all *p* > 0.05). Nevertheless, several differences in the initial clinical presentation were observed. Symptom onset within the first 24 h of life occurred in all Group 1 patients, compared with 77.8% of Group 2 patients. Median peak FiO_2_ was higher in Group 1 (100% vs. 55%), whereas Group 2 patients required longer durations of mechanical ventilation (55 vs. 20 days). Mortality was numerically higher in Group 1 than Group 2 (40.0% vs. 11.1%). None of these differences reached statistical significance, likely reflecting the limited sample size, and the findings should therefore be interpreted with caution.

Treatment was generally empirical; systemic corticosteroids were administered in 64.3% of patients (9/14), with clinical improvement in 5/9, including all 3 who received combination therapy with hydroxychloroquine.

During a median follow-up of 36 months (Q1–Q3: 2–72), mortality occurred in 3 patients (21.4%). Among the three deceased patients, two belonged to Group 1, both carrying biallelic *ABCA3* mutations, and one belonged to Group 2, carrying the *ITGA3* variant.

## 4 Discussion

In this study, a genetic aetiology was identified in 50%, and considerable respiratory morbidity requiring prolonged oxygen support was observed in patients with neonatal onset chILD. Pathogenic or likely pathogenic variants were most frequently detected in *ABCA3* (21.4%) and *NKX2-1* (14.3%). This distribution was consistent with international series reporting a predominance of surfactant-protein-related and NKX2-1-related disease in early-onset chILD (16, 17). The genetic diagnostic yield in our cohort exceeded that of large international series, which may be related to the early neonatal presentation, high rate of consanguinity, and use of comprehensive molecular testing (18). Marked genetic heterogeneity was observed with surfactant protein metabolism disorders representing a major subgroup. Disorders related to *ABCA3, SFTPC, SFTPB,* and *NKX2-1* showed a wide phenotypic range, from severe neonatal respiratory failure to later-onset chronic forms, consistent with previous reports (17, 18). In *NKX2-1*-related disease, neonatal respiratory distress requiring oxygen support was the initial presentation; in some patients, partial clinical improvement was noted over time despite persistent radiological abnormalities (17, 19). Parental consanguinity and familial clustering were common findings in our cohort, in line with previous studies reporting familial aggregation in surfactant protein dysfunction disorders, particularly in *ABCA3-*related disease (20, 21).

Diffuse infiltrates on chest radiography were present in all patients; however, this finding overlaps with many neonatal respiratory conditions and lacks diagnostic specificity (22, 23). HRCT findings were heterogeneous, including ground-glass opacities, septal thickening, and cystic changes, all of which may be seen in both surfactant protein dysfunction disorders and other forms of diffuse lung disease (23). In a subset of patients, imaging findings were compatible with NEHI/PIG-spectrum disease. These conditions may present in infancy with persistent tachypnoea, hyperinflation, and diffuse ground-glass opacities, and generally follow a more favorable clinical course compared with surfactant protein dysfunction disorders (24, 25). In our cohort, imaging findings were used primarily to support clinical suspicion and guide further diagnostic workup, rather than to independently establish a specific diagnosis. Current European Respiratory Society recommendations similarly state that early genetic testing is indicated in neonatal-onset ILD when clinical suspicion is present (6, 26).

Consistent with the pattern observed in this cohort, Group 1 patients showed earlier and more severe initial presentation but shorter ventilatory dependence than Group 2 (7, 26), a pattern also described in some *NKX2-1*-related and other non-surfactant chILD cohorts (16–18). These findings suggest that the underlying etiology may contribute to differences in the initial clinical presentation; however, larger multicenter cohorts are required to confirm these observations (2).

The cohort included a range of diagnoses encompassing NEHI, PIG, surfactant protein dysfunction disorders, and congenital PAP, reflecting the broad aetiological spectrum of neonatal ILD. This diversity underlines the need for a structured, stepwise diagnostic approach that incorporates imaging, bronchoscopy, histopathology when indicated, and comprehensive genetic testing (4, 6, 25, 27). In the patient with congenital PAP, milky BAL fluid prompted targeted *CSF2RA* analysis, illustrating the diagnostic value of BAL in selected clinical settings (28). Lung biopsy was performed in only 2 of 14 patients (14.3%), and a diagnosis was established in both cases. Biopsy was reserved for patients in whom genetic testing and CT findings remained inconclusive. Recent pediatric series have similarly reported that genetic testing and lung biopsy should be considered complementary diagnostic tools in critically ill children with diffuse lung disease (29).

Evidence supporting treatment decisions in childhood ILD remains largely based on case series and expert opinion rather than controlled studies (4, 27). Recent data suggest that treatment response may vary according to the underlying genetic diagnosis in *ABCA3*-related disease, which may inform future therapeutic approaches (30). In our cohort treatment strategies were empirical. Systemic corticosteroids were used in 64.3% of patients, and hydroxychloroquine was used in selected cases, additionally. Given the small numbers, this should be interpreted as exploratory rather than evidence of combination-therapy superiority.

The mortality rate in this cohort was 21.4%, consistent with recent international series of early-onset chILD (18, 31, 32). The relatively low mortality may reflect the genetic composition of the cohort: no *SFTPB* mutations were identified, and *NKX2-1-*related disease, which followed a more stable pattern in all affected patients, was the second most common genetic etiology (16, 17). In contrast, mortality occurred in two of the three infants with ABCA3-related disease in our cohort, consistent with reports linking biallelic ABCA3 mutations to severe neonatal respiratory failure, although the severity spectrum for this gene can be broad (7); complete SFTPB deficiency has similarly been associated with severe neonatal respiratory failure and early mortality in large registry data (31). Both deaths in Group 1 occurred in the two patients with biallelic *ABCA3* mutations who were too clinically unstable to undergo chest CT during the neonatal period and were diagnosed by genetic testing alone; this observation is consistent with the known severity of *ABCA3-*related surfactant dysfunction (1, 7, 20). The single death in Group 2 occurred in the patient with the *ITGA3* variant; given the limited functional evidence supporting a causal role for *ITGA3* in this phenotype, the contribution of this variant to the fatal outcome remains uncertain. The relatively low overall mortality in this cohort may partly reflect its genetic composition rather than diagnostic yield *per se*, as discussed above.

Delayed referral in preterm infants deserves particular attention. In this study, preterm infants were referred to the pediatric pulmonology department at a later median time point than term infants. Although this difference did not reach statistical significance, the clinical implication is relevant: in preterm neonates, persistent respiratory failure is commonly attributed to RDS or evolving BPD, and the possibility of an underlying diffuse lung disorder may not be considered until later in the clinical course. In particular, *ABCA3-*related disease may be clinically indistinguishable from classical RDS in preterm infants, and diagnostic delay is likely when surfactant therapy fails to produce sustained improvement (20, 26, 32, 33). These observations suggest that heightened clinical awareness of chILD is warranted in preterm infants who fail to wean from respiratory support or who require repeated surfactant administration without sustained benefit, even in the absence of a family history of ILD.

This study has several limitations. Its retrospective, single-center design, small sample size, and high rate of consanguinity may limit generalizability. Group 2 encompassed a clinically and etiologically heterogeneous set of diagnoses, grouped together due to sample size constraints; this heterogeneity should be considered when interpreting between-group comparisons, as it may obscure diagnosis-specific differences within this category. Given the small subgroup sizes, our findings on treatment response and genotype-specific prognosis should be considered exploratory rather than confirmatory. The absence of *SFTPB* and *SFTPC* mutations may also have influenced the observed mortality (31, 34). In addition, two genetic findings should be interpreted cautiously. The diagnosis of PAP was established based on the clinical presentation, characteristic BAL findings, and radiological features; the identified heterozygous *CSF2RA* variant, in the absence of functional validation, cannot be considered definitively disease-causing. One patient with a homozygous *TNFRSF13B* variant was included based on compatible clinical, radiologic, and treatment-response findings. Although the exact contribution of this variant to the pulmonary phenotype remains uncertain, no alternative diagnosis explaining the diffuse lung disease was identified. Despite these limitations, our cohort provides real-world data on neonatal ILD from a population with high consanguinity and highlights the marked clinical heterogeneity of these disorders (35). Further multicenter prospective studies are needed to better define genotype–phenotype relationships and long-term outcomes. Importantly, repeated surfactant administration and failure to wean from respiratory support may represent practical clinical red flags prompting early genetic evaluation for neonatal chILD, particularly when no alternative explanation for persistent respiratory failure is identified.

## 5 Conclusion

Given that preterm infants made up half of this cohort, our findings suggest that chILD should remain part of the differential diagnosis, and be approached with a broad, multimodal perspective — clinical, radiological, and genetic — in neonates with unexplained or refractory respiratory failure, regardless of gestational age. Neonatal-onset chILD should be considered in infants with persistent respiratory failure that cannot be explained by common neonatal conditions. In our cohort, a genetic diagnosis was established in half of the patients, with *ABCA3* and *NKX2-1* variants being the most frequent genetic findings. Although between-group comparisons did not reach statistical significance, reflecting the limited sample size, Group 1 patients showed numerically earlier symptom onset and higher peak oxygen requirements than Group 2 patients — a pattern that warrants confirmation in larger cohorts. Both fatalities in Group 1 occurred in patients with biallelic *ABCA3* mutations consistent with the known severity of this genotype in the existing literature. Repeated surfactant administration or failure to wean from respiratory support should prompt consideration of chILD and early genetic evaluation. Particular attention may be warranted in preterm infants, in whom persistent respiratory failure is often attributed to respiratory distress syndrome or bronchopulmonary dysplasia, potentially delaying consideration of chILD.

## Data Availability

The datasets generated and/or analyzed during the current study are not publicly available due to patient confidentiality restrictions but are available from the corresponding author upon reasonable request.
